# Concordance of Solid Organ Biopsy Diagnoses With Hospital Autopsy and the Contribution of Biopsies to Death

**DOI:** 10.7759/cureus.33889

**Published:** 2023-01-17

**Authors:** David S Priemer, Joseph M Curran, Carrie L Phillips, Oscar W Cummings, Romil Saxena

**Affiliations:** 1 Department of Defense/ Uniformed Services University Brain Tissue Repository, Henry M Jackson Foundation, Bethesda, USA; 2 Pathology, Uniformed Services University of the Health Sciences, Bethesda, USA; 3 Pathology, The Broward County Office of Medical Examiner & Trauma Services, Ft. Lauderdale, USA; 4 Pathology, Indiana University School of Medicine, Indianapolis, USA; 5 Pathology, Emory University School of Medicine, Atlanta, USA

**Keywords:** biopsy, quality assessment, therapeutic complications, kidney biopsy, lung biopsy, liver biopsy, autopsy

## Abstract

Biopsies of the liver, lung, and kidney are performed for many indications, including organ dysfunction, mass lesions, and allograft monitoring. The diagnosis depends on the sample, which may or may not be representative of the lesion or pathology in question. Further, biopsies are not without risk of complications. Autopsies are a resource for assessing the accuracy of biopsy diagnoses and evaluating possible complications. Herein, we aimed to compare liver, lung, and kidney biopsy diagnoses with those from autopsies conducted soon after the procedure and to assess the contribution of biopsy to mortality. A 28-year search of our database identified 147 patients who were autopsied after dying within 30 days of a liver, lung, or kidney biopsy. The concordance of the biopsy diagnosis with the autopsy findings was determined. Finally, medical records were reviewed to determine the likelihood that a biopsy contributed to the patient's death. The contribution of the biopsy to death was categorized as "unlikely," "possible," or "probable." Overall concordance between biopsy and autopsy diagnoses was 87% (128/147), including 95% (87/92), 71% (32/45), and 90% (9/10) for liver, lung, and kidney biopsies, respectively. Concordance was lower for biopsies of suspected neoplasms versus non-neoplastic diseases. Lung biopsy concordance was higher for wedge biopsy versus needle or forceps biopsy. A biopsy was determined to at least "possibly" contribute to death in 23 cases (16%). In conclusion, an autopsy is an important tool to validate liver, lung, or kidney biopsy diagnoses. Confirmation of biopsy diagnoses via post-mortem examination may be particularly valuable when patients die soon after the biopsy procedure. Furthermore, an autopsy is especially useful when patients die soon after a biopsy in order to determine what role, if any, the procedure played in their deaths. Though biopsy complications are uncommon, a biopsy may still contribute to or precipitate death in a small number of patients.

## Introduction

Biopsies of the liver, lung, and kidney are routinely performed for a variety of indications, including the assessment of organ dysfunction, the characterization of mass lesions, and the monitoring of allograft function. In each case, the biopsy diagnosis forms the basis of clinical management. However, the diagnosis depends on the area sampled, which may or may not be representative of the organ or lesion as a whole. The diagnosis also depends on the size and quality (adequacy) of the sample. When available, autopsies are an invaluable resource for evaluating the accuracy and quality of diagnoses rendered on biopsy specimens of solid organs. However, while a number of studies assessing the accuracy of liver, lung, and kidney biopsies as compared to surgical resection specimens and/or clinical course exist [1-4], literature regarding the utility of post-mortem examination as a quality assessment tool for biopsy procedures is lacking. This is particularly true for biopsies taken for non-neoplastic disease without a mass lesion. Also, although generally safe (the reported "major" complication rates of transjugular liver biopsies, transthoracic lung biopsies, and percutaneous renal biopsies are <2%, <5%, and <3%, respectively [5-11]), liver, lung, and kidney biopsies are associated with a number of possible complications, including death. Autopsies, therefore, represent an important tool for assessing the contribution of biopsy procedures to patient mortality.

Herein, we aimed to compare liver, lung, and kidney biopsy diagnoses with those from autopsies conducted soon after the procedure and to assess the contribution of biopsy to mortality. The data presented in this study were also presented as a poster at the 107th annual meeting of the United States and Canadian Academy of Pathology in San Antonio, TX, USA, and therein selected for the Autopsy Award by the Association of Directors of Anatomic and Surgical Pathology.

## Materials and methods

A retrospective search of the computerized pathology laboratory information system at the Indiana University School of Medicine, Department of Pathology and Laboratory Medicine, was performed over a 28-year period to identify patients who were autopsied after dying within 30 days of either a forceps, needle, or wedge biopsy of the liver, lung, or kidney. This was accomplished by identifying patients within the system who had both a surgical pathology accession number for the aforementioned qualifying biopsy and an accession number for an autopsy procedure and including only those who died within 30 days of the biopsy procedure. Patient biopsy reports and autopsy reports were then reviewed to establish the accuracy of the biopsy diagnosis. In addition, patient autopsy reports and, when accessible, their electronic medical records were also reviewed retrospectively to determine the contribution of the biopsy procedure to death. The contribution of a biopsy to death was subjectively categorized as "unlikely" (biopsy did not contribute to death), "possible" (a biopsy may have contributed to death), or "probable" (biopsy had a direct or near-direct contribution to death).

This study, which involved only deceased individuals, did not meet the criteria for human subject research and accordingly did not require review by the Institutional Review Board at the Indiana University School of Medicine. This study involved only deceased individuals for whom the legal next-of-kin consented to postmortem examination at the Indiana University School of Medicine at the time of death, which included consent for the utilization of materials in education and research.

## Results

Patient and clinical data

A total of 147 patients met the inclusion criteria, including 87 males and 60 females. The average patient age was 49 years (ranging from two weeks to 78 years; median: 52 years) in the liver biopsy group; 52 years (range: five weeks to 78 years; median: 58 years) in the lung biopsy group; 52 years (range: 29 to 77 years; median: 55 years) in the kidney biopsy group; and 50 years (range: two weeks to 78 years; median: 54 years) overall. There were 78 patients (53%) whose age was 55 years or less. The time interval between biopsy and death averaged nine days in the liver biopsy group (range: 0-29 days; median: 7.5 days), 10 days in the lung biopsy group (range: 0-30 days; median: nine days), 11 days in the kidney biopsy group (range: one to 29 days; median: 7.5 days), and 10 days overall (range: 0-30 days; median: eight days). Eleven (7%) of the patients underwent a biopsy for what was confirmed to be a neoplastic disease, and the remaining 136 (93%) underwent a biopsy for what was confirmed to be a non-neoplastic disease.

Concordance

The concordance data is shown in Table 1. The concordance between biopsy and autopsy diagnoses was 95% (87/92 cases) in the liver group, 71% (32/45 cases) in the lung group, 90% (9/10 cases) in the kidney group, and 87% (128/147 cases) overall. The overall concordance in neoplastic cases was 72% (8/11 cases) and 88% (120/136 cases) in non-neoplastic cases. The concordance in cases with core needle biopsies of the liver was 95% (91/95 cases) and 86% (6/7 cases) for cases in which wedge biopsies of the liver were performed. Data regarding the precise method by which a core needle biopsy of the liver was obtained (e.g., transjugular biopsy, endoscopic ultrasound-guided biopsy, transcutaneous biopsy, operative) was not readily available in a large proportion (approaching 60%) of the cases involving core needle biopsies of the liver, particularly in older cases whose digital records were limited; therefore, differences between these methods of the liver biopsy were not systematically examined. The concordance in cases with needle or forceps biopsies of the lung was 64% (16/25 cases) and 80% (16/20) for cases in which a wedge biopsy of the lung was performed. All cases in the kidney group involved core biopsies.

**Table 1:** Concordance of diagnoses between autopsy and liver, lung, and kidney biopsies Comparison by organ type and assessment for either neoplastic or non-neoplastic disease

Organ	Concordance: neoplastic disease (n=11)	Concordance: non-neoplastic disease (n=136)	Concordance: overall (n=147)
Liver	100% (7/7 cases)	94% (80/85 cases)	95% (87/92 cases)
Lung	25% (1/4 cases)	76% (31/41 cases)	71% (32/45 cases)
Kidney	n/a (0 cases)	90% (9/10 cases)	90% (9/10 cases)
All	72% (8/11 cases)	88% (120/136 cases)	87% (128/147 cases)

Biopsy contribution to death

Of the 147 cases, it was determined that the biopsy procedure had a "probable" contribution to death in 10 cases (7%), and a "possible" contribution to death in 13 cases (9%), amounting to 23 total cases (16%) in which the biopsy procedure may have influenced death (Table 2). The average patient age in these 23 cases was 46 years (range: two weeks to 77 years; median: 49 years) and included 14 patients (61%) whose age was 55 years or less. The average patient age in the remaining 124 cases (the "unlikely" group) was 50 years (range: 22 weeks to 78 years, median: 55) and included 64 patients (52%) whose age was 55 years or less. The average intervals between biopsy and death were four days (range: 0 to nine days; median: three days) in the "probable" group, five days (range: 0 to 24 days; median: three days) in the "possible" group, and 11 days (range: 0 to 30 days; median: nine days) in the "unlikely" group.

**Table 2:** Contribution of the biopsy procedure to the patient's death "Probable": death was influenced by or occurred as a result of a biopsy; "Possible": a biopsy may have contributed to death; "Unlikely": A biopsy did not appear to contribute to death.

Contribution to death	Liver biopsy (n=92)	Lung biopsy (n=45)	Kidney biopsy (n=10)	Overall (n=147)
"Probable"	7% (6/92 cases)	7% (3/45 cases)	10% (1/10 cases)	7% (10/147 cases)
"‘Possible"	10% (9/92 cases)	7% (3/45 cases)	10% (1/10 cases)	9% (13/147 cases)
"Unlikely"	84% (77/92 cases)	87% (39/45 cases)	80% (8/10 cases)	84% (124/127 cases)

**Figure 1:**
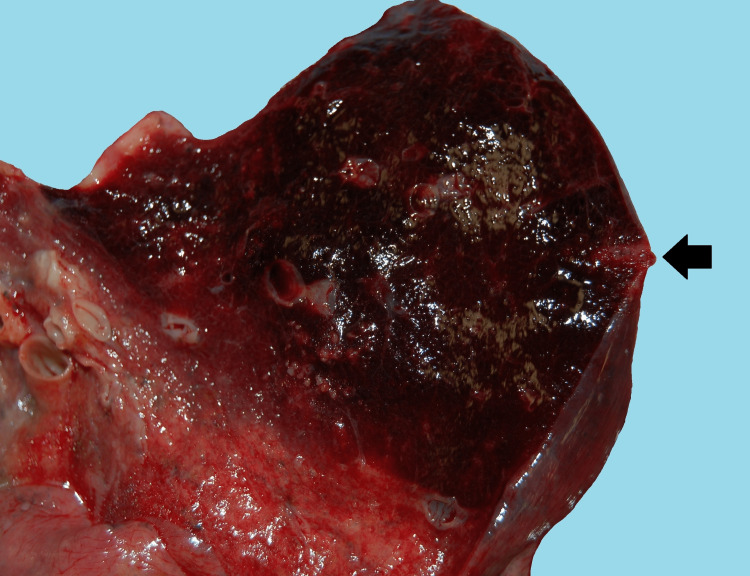
A case example of a lung biopsy as a "probable" contributor to death Laceration and intraparenchymal hemorrhage at the site of a right lung transthoracic biopsy (arrow) were performed approximately four days prior to death. This finding was associated with a 3L hemothorax. The 74-year-old patient was admitted for anticoagulation therapy for pulmonary emboli, and a biopsy was performed for a suspicious lung nodule seen in imaging studies. The biopsy did not reveal evidence of malignancy, and post-mortem examination of the lung did not identify a mass lesion.

**Figure 2:**
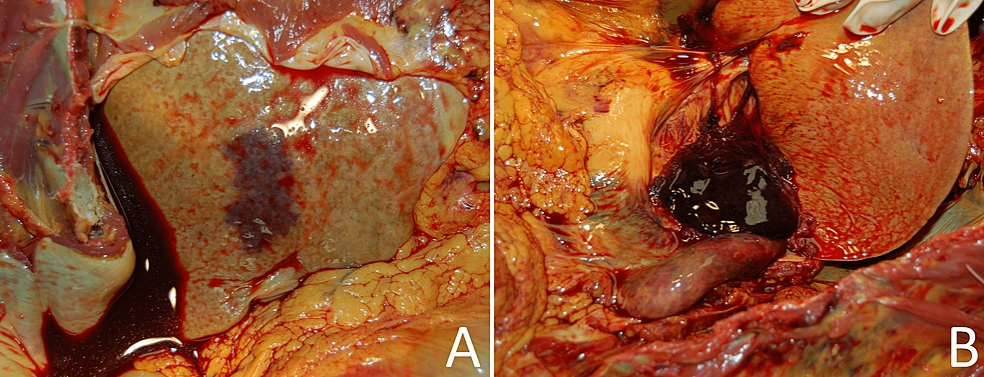
A case example of a liver biopsy as a "probable" contributor to death A subcapsular hematoma (A), which extended to porta hepatis (B), following a liver needle biopsy performed one day prior to death. This finding was associated with a 1L hemoperitoneum. The 62-year-old patient was admitted with a right lung mass and widespread replacement of liver parenchyma by tumor, which was confirmed to be metastatic non-small cell carcinoma by transgastric liver biopsy and autopsy.

Eight of the 10 cases in which a "probable" designation was assigned involved significant bleeding as the main complication of the biopsy procedure (Figures 1, 2).

A summation of clinical information, biopsy findings, and autopsy findings of the cases in which biopsy "probably" contributed to death is provided in Table 3.

**Table 3:** Biopsy as a "probable" contributor to death Clinical and autopsy summaries of cases FNA: fine needle aspiration

Age/sex	Biopsy site and type	Biopsy indication	Biopsy diagnosis	Clinical complications after biopsy	The time between biopsy and death	Autopsy–biopsy concordance	Autopsy findings relating to the biopsied organs
39y/F	Liver, core	Elevated liver enzymes in the setting of hepatitis C and alcohol abuse	Chronic hepatitis with cirrhosis (stage 4/4)	Markedly decreased hemoglobin concentrations	3 days	Yes	3L hemoperitoneum and 210g hematoma in the right upper quadrant
55y/F	Liver, core	Elevated liver enzymes in the setting of a vulvar rash	Multifocal punctate necrosis with viral inclusions, consistent with herpes virus hepatitis.	Episodic hypotension	2 days	Yes	3.5L hemoperitoneum and hematoma on anterior surface of liver.
53y/F	Liver, core	Elevated liver enzymes; concern for graft-versus-host disease	Hemosiderosis, no evidence of graft versus host disease	Episodic hypotension	3 days	Yes	1.5 liters of bloody ascites; 3.0 x 2.0 cm intraparenchymal hematoma with porta hepatis extension.
30y/M	Liver, core	Elevated liver enzymes in the setting of fever and rash	Features most consistent with hemophagocytic lymphohistiocytosis	Loss of responsiveness, arrhythmia, and fluid accumulation in the abdomen	<1 day	Yes	2.0 liters of acute hemoperitoneum.
62y/M	Liver, core	Widespread involvement of the liver and lung by a tumor of unknown primary	Poorly differentiated adenocarcinoma, most consistent with metastatic carcinoma from a primary lung tumor	Worsening hypotension, bradycardia, and cardiac arrest.	1 day	Yes	1L hemoperitoneum, subcapsular hematoma
3w/M	Liver, wedge	Abnormal liver function in the setting of prematurity, birth defects, and maternal herpes simplex infection.	Marked lobular collapse with regenerative changes. Increased iron stores.	Deteriorated general clinical status	9 days	Yes	50 mL of hemoperitoneum with intra-abdominal clots and multiple intraparenchymal hemorrhages with organization
74/F	Lung, transthoracic biopsy, and fine needle aspiration	Suspected neoplasm (lung nodule on imaging) in the setting of anticoagulation therapy for pulmonary emboli	No evidence of malignancy, mild interstitial fibrosis, or chronic inflammation	Hemoptysis	4 days	Yes	3L right-sided hemothorax with 1650 g of clotted blood, sourced from a pleural laceration at the FNA/biopsy site
77/F	Lung, transbronchial biopsy	Basilar lung infiltrates and fever	Interstitial fibrosis and non-specific chronic inflammation.	Acute hypotension and hypoxia during the bronchoscopy procedure, requiring mechanical ventilation, and the eventual development of acute respiratory distress syndrome	4 days	Yes	Panlobar, severe diffuse alveolar damage in a background of focal organizing pneumonia and focal acute bronchopneumonia.
44/M	Lung, transbronchial biopsy	Diffuse interstitial infiltrates in the setting of dermatomyositis and numerous chronic occupational exposures	Nonspecific reactive lung changes	Pneumo-mediastinum	9 days	Yes	Panlobar, severe diffuse alveolar damage.
65/M	Kidney, needle	Acute renal failure in the setting of liver failure.	Acute tubular necrosis, IgA nephropathy, and arterionephrosclerosis	Rapid clinical decline and multiorgan failure	3 days	Yes	“Very large” left perirenal and retroperitoneal hematoma extending from the left hemidiaphragm to the pelvis

## Discussion

The Latin phrase "ibi mortui vivos docent", translated as "Let the dead teach the living," is posted, appropriately, in many autopsy suites throughout the world. Further, the ancient Greek derivation of the modern word "autopsy" literally translates to "seeing with one’s own eyes" (being formed from the ancient Greek autos, or "self," and ópsis, or "sight," and together as "autopsía"), and as such, the autopsy has been regarded as the "gold standard" for quality assessment of clinical observations [12,13]. The autopsy is an exceedingly useful medical procedure that affords direct examination of the decedent’s body in its entirety through dissection. Discrepancies between clinical diagnoses and findings at autopsy were recognized and described as early as 1912, and the rates of discordance have decreased only slightly over time despite major technological advancements in clinical medical diagnostics [14-17]. These stagnant rates indicate that the autopsy should remain an essential part of the healthcare system and not be considered merely as an occasional adjunct. Further, the autopsy should be viewed as the definitive investigative procedure for assessing the accuracy and risk of any diagnostic test. However, most published research to date investigating the autopsy as a quality assurance tool for specific types of clinical tests focuses on comparisons to medical imaging [18,19].

This study aimed to determine the correlation of pre-mortem liver, lung, and kidney biopsy diagnoses with post-mortem findings from autopsies that took place shortly after the biopsy procedure, more specifically those involving deaths that occurred within 30 days of the biopsy procedure. This short timeframe ensures that the biopsy findings will parallel or otherwise closely reflect the findings at autopsy. In addition to evaluating the concordance of diagnoses, we also assessed the likelihood that the biopsy procedure contributed to the death of the patient. Our search of autopsied patients who received either a needle, forceps, or wedge biopsy of the liver, lung, or kidney within 30 days of death spanned a period of 28 years and identified 147 patients, with an average interval between biopsy and death of 10 days.

While we showed in our study that the majority of these biopsies were accurate, we found an overall discrepancy rate of 13% between the biopsy and autopsy findings. The discrepancy rate was highest in lung biopsies (29%), followed by kidney biopsies (10%), and was the lowest in liver biopsies (5%). The discrepancy rate was higher in biopsies taken for suspected neoplasia (28%), particularly of the lung, than in biopsies for non-neoplastic disease (12%). The difference is likely accounted for by a sampling error, given that biopsies for suspected neoplasia are targeted toward a focal lesion, and within that focal lesion there may or may not be regions of little or no tissue viability (e.g., tumor necrosis); this is in contrast to biopsies for non-neoplastic disease, which do not necessarily rely on a precise biopsy site. The discrepancy rate for non-neoplastic lung biopsies was also considerably higher than that for non-neoplastic diseases of the liver and kidney, though the rate improved when wedge biopsies (20%) were taken as opposed to much smaller needle or forceps biopsies (36%), which are susceptible to being non-representative. Interestingly, the discrepancy rate was higher among liver wedge biopsies versus core biopsies; we suggest that this is most likely due to the sample size (i.e., the small number of liver wedge biopsies in the cohort); however, it is also possible that liver wedge biopsies may provide an inadequate amount of parenchyma deep to the subcapsular region necessary for optimal pathologic evaluation. This is a consideration for future studies. Unsurprisingly, the overall discrepancy rate of 13% in liver, lung, and kidney biopsies in this study is smaller than the overall discrepancy rate of 27% between FNA diagnoses and autopsy findings highlighted by another recent study [20]. Regardless, however, our data further highlight the diagnostic discrepancies that continue to exist between pre- and post-mortem diagnoses in modern medicine. Given that patients who die soon after a liver, lung, or kidney biopsy procedure often cannot have that diagnosis confirmed via excisional material, treatment, and/or long-term clinical observation, our data also further emphasize that autopsy is an important quality assessment tool.

Our review of records revealed that biopsies of the liver, lung, and kidney generally do not result in death. The literature also associates these biopsies with a low risk of major complications or death [5-11]. However, we determined that in 10 of the 147 cases, the biopsy procedure had a "probable" contribution to death, in that death was strongly influenced by or directly attributed to the biopsy procedure. This was in addition to 13 cases in which the biopsy had a "possible" contribution to death, yielding a total of 23 cases (16%) in which the biopsy procedure may have contributed to the patient’s death. The most common complicating factor across all three organ systems was overwhelmingly hemorrhage. For example, cases in which a kidney biopsy contributed to death involved massive retroperitoneal hemorrhage in one case and a large perinephric hematoma in another. In cases where liver biopsies contributed directly to death ("probable"), autopsies showed large hematomas in or around the liver, massive free blood within the peritoneal cavity (up to 3.5 liters in one case), or a combination of both. Coagulopathy at the time of the biopsy is the most significant risk factor in the development of these complications [9,10]. In our study, complications of lung biopsy procedures contributing to death varied from pneumomediastinum to hemothorax to complications necessitating intubation with subsequent development of acute respiratory distress syndrome. Ultimately, we demonstrate that, although ours is a selective autopsy sample, which cannot offer inferences about the overall prevalence of complications, there is a small proportion of cases in which a biopsy procedure of the lung, liver, or kidney may iatrogenically contribute to and precipitate death. It is critical to perform autopsies in suspected cases.

The overall average interval between biopsy and death in our entire cohort was 10 days. Among the 23 cases in which a biopsy "probably" or "possibly" contributed toward death, the average interval between a biopsy and death was four and five days, respectively, and in both groups, the interval did not exceed nine days. This is in comparison to the cases in which a biopsy was determined to be non-contributory to death, which had an average interval of 11 days, with many deaths occurring later. Interestingly, though not unexpectedly, this suggests that the sooner a death occurs after a liver, lung, or kidney biopsy, the more likely the procedure might have contributed to the death.

## Conclusions

The importance of the autopsy cannot be overstated. The accuracy of a biopsy diagnosis often cannot be ascertained when a patient dies soon after the procedure unless an autopsy is performed. Similarly, the contribution of the biopsy procedure to death cannot be determined without an autopsy on these patients. Our results show that the autopsy can be used as a valuable quality assessment tool to validate the accuracy of the biopsy diagnosis of the liver, lung, and kidney; we further demonstrate that these biopsy diagnoses are largely accurate. The discrepancy rate was highest in lung biopsies as compared to liver and kidney biopsies, but it improved when larger wedge biopsies of the lung were obtained. Further, our results show that while liver, lung, and kidney biopsies generally do not cause serious complications, there is a small proportion of cases in which a biopsy procedure may contribute to or precipitate death. This study, therefore, is also a call for continued dialogue into what impact the utilization of autopsies, as an essential component of patient outcome surveillance, has on the overall morbidity within the healthcare system.
